# Thermostable Cellulases from the Yeast* Trichosporon* sp.

**DOI:** 10.1155/2019/2790414

**Published:** 2019-04-17

**Authors:** Hanane Touijer, Najoua Benchemsi, Mohamed Ettayebi, Abdellatif Janati Idrissi, Bouchra Chaouni, Hicham Bekkari

**Affiliations:** ^1^Sidi Mohamed Ben Abdellah University, Faculty of Sciences Dhar El Mahraz, Laboratory of Biotechnology, P.O. Box 1796, 30003 Fez-Atlas, Morocco; ^2^Sidi Mohamed Ben Abdellah University, Faculty of Sciences Dhar El Mahraz, Biodiversity, Bioenergy & Environment Research Group (BBE), P.O. Box 1796, 30003 Fez-Atlas, Morocco; ^3^Sidi Mohamed Ben Abdellah University, Faculty of Sciences & Techniques Saiss, Laboratory of Ecology and Environment, P.O. Box 1796, 30000 Fez, Morocco; ^4^Laboratory of Plant and Microbial Biotechnology, Biodiversity and Environment, Faculty of Sciences, University Mohamed V in Rabat, 10000 Rabat, Morocco

## Abstract

**Objectives:**

Identification of cellulolytic microorganisms is of great interest to the hydrolysis of cellulosic biomass. This study focuses on the identification of cellulolytic yeasts and the optimization of cellulase activities produced by the best performing isolate.

**Results:**

30 cellulolytic yeast isolates were selected. Enzymes produced by an isolate from the* Trichosporon* genus showed the property to hydrolyze different substrates: carboxymethyl cellulose (CMC), cellulose fiber, and filter paper (FP). The optimum measured temperature was 55°C for CMCase and 60°C for FPase. The optimal pH was 5 for CMCase and 4 to 6 for FPase. The effect of the substrates concentration showed that the best activities were obtained at 100 mg/mL CMC or FP. The highest activities were 0.52 for the CMCase and 0.56 for the cellulase fiber at 10 min incubation, 0.44 IU/mL at 15 min incubation, and 24 h FPase preincubation.

**Conclusion:**

Cellulases produced by the studied yeast are capable of hydrolyzing soluble and insoluble substrates at elevated temperatures and at a wide pH range. They are considerable interest in the production of fermentable sugars from lignocellulosic substrates.

## 1. Introduction

Cellulose is the most important constituent of the plant cell wall [1]. This polymer is widely used in many industry sectors such as agrifood, paper mill, and bioethanol production [2]. Interestingly, bioethanol made from cellulosic biomass attracts lots of attention as a biofuel, with economic advantages and minimal impact on the environment [1]. Bioethanol production requires four major steps: biomass pretreatment, cellulose hydrolysis, fermentation, and distillation [3]. Cellulose hydrolysis to glucose could be performed by chemical, physical, or enzymatic processes. Enzymatic hydrolysis is the most used in the industry because of its high yields with fewer unwanted side products [4]. It is achieved by a simultaneous action of three enzymes: endo-*β*-glucanase (EC 3.2.1.4), exo-*β*-glucanase (EC 3.2.1.91), and *β*-glucosidase (EC 3.2.1.21) [5]. Fungi and bacteria are potential producers of cellulase enzymes [6, 7]. Nowadays, yeast cellulases gain interest, as they are active in a wide pH range and at high temperatures [8, 9]. Indeed, yeasts can produce thermostable cellulase, undenatured at temperatures greater than or equal to 70°C [10, 11]. They can be used in the processes of saccharification of lignocellulose into fermentable sugars for the production of bioethanol.

This study aims to isolate yeast strains from the gastrointestinal tract of the coprophage “*Gymnopleurus sturmi*” capable of producing cellulases and then to optimize the measurement conditions of cellulase activities.

## 2. Materials and Methods

### 2.1. Microorganisms and Culture Conditions

The selection of cellulose isolates in yeasts was determined by growing them on Mandels and Weber medium (MW, 1969), which was based on the carboxymethyl cellulose (CMC) as a unique source of carbon and energy. The incubation was performed at 37°C for 48 to 72 hours. The cellulase activity was revealed by the addition of a solution of 1% Congo red, which binds to the polymers of cellulose. After 15 to 20 minutes, the dishes were washed with a solution of NaCl (1M). The rise/appearance of a light halo around the colonies indicates that the incorporated CMC in the medium was degraded by the cellulase secreted by the isolates; the latter are called “cellulase +”.

### 2.2. Molecular Characterization

The molecular characterization was performed by carrying out sequencing of DNA ITS region. This region was amplified with ITS1 and ITS4 pair of primers. A 25 *μ*L of the PCR reaction mixture was prepared with 0.2 *μ*l of Taq (0.01U/*μ*l), 0.625 *μ*l of each primer, 1 *μ*l of dNTPs (0.2mM), 5 *μ*l of Buffer (1X), 1.75 *μ*l MgCl2 (1.5mM), 2 *μ*l d'ADN, and 13.8 *μ*l of water ultrapure. Amplification reaction was performed in the TECHNE thermocycler. The sequences were generated by ABI PRISM 3130XL (Applied Biosystems) as genetic analyzer. Then the results were processed by the BioEdit software and the nucleotide sequence was analyzed using the Basic Local Alignment Search Tool (BLAST).

### 2.3. Evaluation of Cellulase Enzymes Production by* Trichosporon *sp

The characterization and optimization of cellulase activities are carried out on a yeast isolate that belongs to the genus* Trichosporon*, which was the most powerful with regard to the hydrolysis halo of CMC diameter, in a qualitative test. The MW medium was used for the production of these enzymes. The inoculum of the studied yeast was added to the culture medium. Then, the mixture was incubated at 37°C for 4 days, the cell suspension was centrifuged (6000g, 10 min), and the recovered supernatant was used as an enzymatic medium for the measurement of cellulase activities.

### 2.4. Measurement of Cellulase Activities

For CMCase and cellulase fiber activity, 0.5 mL of cellulosic substrates, CMC, or 1% (w/v) cellulose fiber, dissolved in sodium acetate buffer (0.1 M, pH 4.8), was preincubated for 10 min at 50°C. Then 0.5 mL of the enzyme medium was added, and the mixture was incubated at 50°C for 30 min.

For FPase activity, 50 mg Whatman No. 1 filter paper (1 × 6 cm pieces), added to 1 mL acetate buffer (0.1 M, pH 4.8), was preincubated at 50°C for 10 min. Then, 0.5 mL of the enzyme medium was added. The mixture was incubated at 50°C for 60 min.

The reduction of the sugar released into the reaction mixture was determined by the dinitrosalicylic acid (DNS) method [12].

### 2.5. Effects of the Temperature and pH on CMCase and FPase Activities

The impact of temperature on the activity of the enzymes CMCase and FPase was evaluated at a 4.8 pH and incubation at different temperatures ranging from 20 to 100°C of the reaction mixtures. The study of the effect of pH on the variation of the enzymatic activities was carried out by incubating the reaction mixtures at 50°C, over a pH ranging from 3 to 9.

### 2.6. Effect of Substrate Concentration on CMCase and FPase Activities

The cellulase activity variation in terms of the substrate concentration was tested for different CMC concentrations, ranging from 0 to 100 mg/mL and for FP quantities ranging from 10 to 100 mg under the following conditions (pH 4.8, T° at 50°C).

### 2.7. Kinetics of CMCase and FPase

The kinetic of the studied enzymes was determined by measuring the concentration of glucose released during the hydrolysis of the various substrates (CMC, FP) at incubation times ranging from 10 to 60 min.

The optimization of the hydrolysis activity of the filter paper was carried out after preincubation (0, 30, 60 min, and 24 h) of the filter paper in the acetate buffer (0.1M, pH 4.8).

### 2.8. Calculation Method

The released glucose concentration was used to calculate the cellulase enzyme rates after hydrolysis of the cellulosic substrates. The international unit (IU) of cellulase activity is defined as the amount of enzyme which releases 1 *μ*mol of glucose per minute [13].

(1) Cellulase  activity  IU/mL=Glucose  released  μmole0.5 mL  of  enzymatic  medium×Incubation  time  min

The protein concentration was measured according to the method of Bradford (1976). The specific activity was determined by the ratio of the enzyme's catalytic concentration to the protein concentration (IU/mg protein).

(2) $$\begin{array}{c} Specific\;\;activity\;\;IU/mg=\frac{Cellulase\;\;activity\;\;IU/mL}{\left[protein\right]\;\;mg/mL} \end{array}$$

## 3. Results and Discussion

### 3.1. Screening of Yeasts Isolates for Cellulases Production

Isolation of yeasts from the digestive tract of the coprophage «*gymnopleurus sturmi*» resulted in a collection of 55 isolates, 30 of which was cellulosic. Among these isolates, an isolate with very high hydrolysis activity has been optimized for its CMCase and FPase activities. The DNA sequence analysis of the ITS region helped us to identify the isolate that was from the genus* Trichosporon.*

### 3.2. Hydrolysis of Different Cellulosic Substrates

**Figure 1:**
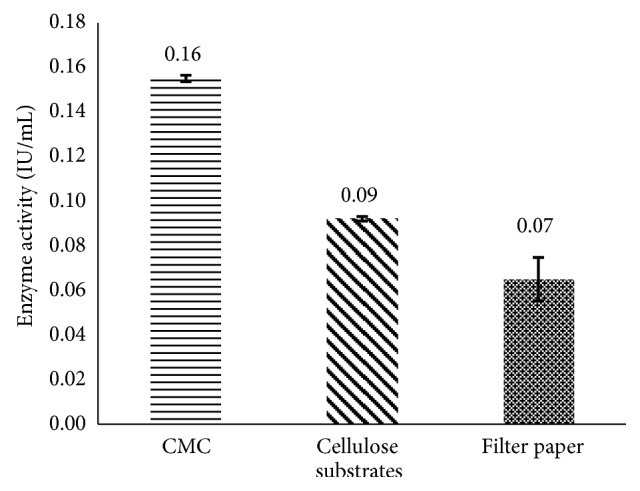
Hydrolysis of cellulosic substrates.

The cellulases produced by the selected yeast were made in evidence by the incubation of the enzyme medium with different substrates, namely, CMC, cellulose fiber, or filter paper. Results of Figure 1 show that the yeast* Trichosporon *sp. has a CMCase activity, fiber cellulase, and FPase of 0.16 IU/mL, 0.09 IU/mL, and 0.07 IU/mL, respectively. Hence, the enzymes produced are capable of hydrolyzing soluble CMC and insoluble cellulose substrates such as cellulose fiber and filter paper. Studies on* Trichosporon laibachii* yeast have shown that despite its CMCase activity, this strain is unable to hydrolyze insoluble substrates like cellulose fiber and filter paper [9]. Similar results are also observed on fungi, as Lucas et al. (2001) detected no cellulase able to hydrolyze insoluble substrates.

The characterization of CMCase and FPase activities was carried out to determine the optimal conditions for measuring these activities, namely, temperature, pH, substrate concentration, and the reaction time.

### 3.3. Effect of Temperature on CMCase and FPase Activities.

**Figure 2:**
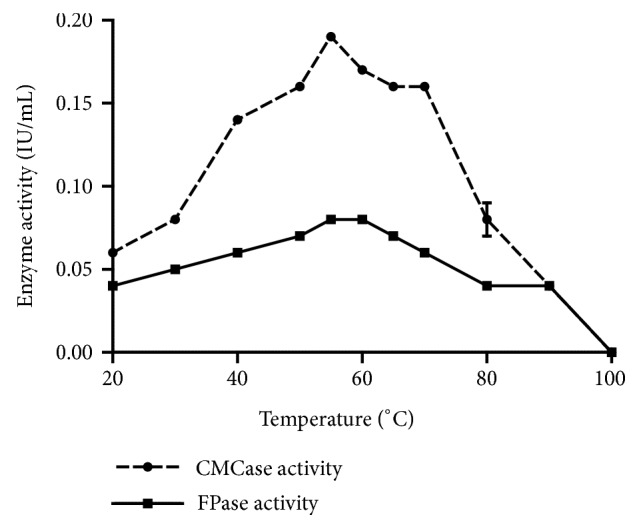
Effect of temperature variation of incubation on CMCase and FPase activities (pH 4.8, 10mg/mL CMC, and 50 mg/mL filter paper).

Results of Figure 2 show that CMCase and FPase are active at temperatures ranging from 20 to 90°C, with optimal temperature at 55°C: the CMCase activity was 0.19 IU/mL and FPase activity was 0.08 IU/mL at 60°C. The residual CMCase and FPase activities were at 44.44% and 50%, respectively, even at a higher temperature as 80°C. Indeed, the strain of the studied yeast* Trichosporon *sp. is able to hydrolyze the cellulosic substrates even at high temperatures. In a similar work on fungi, such as* Aspergillus niger* and* Penicillium decumbens,* the maximum CMCase activity is observed at 50°C [14]. On the other hand, Nawaz S. et al. [15] found that the optimal temperature of the CMCase of the fungus* Trichoderma harzianum* is only 30°C

### 3.4. Effect of pH on CMCase and FPase Activities

**Figure 3:**
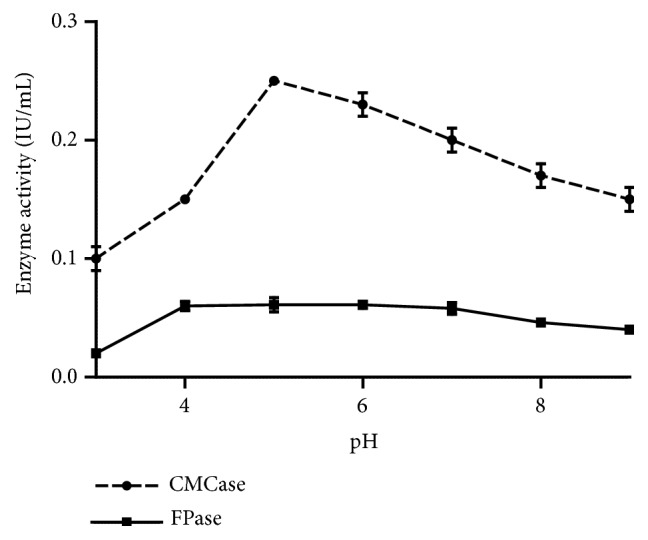
Effect of pH on CMCase and FPase activity (temperature 50°C, 10 mg/mL CMC, and 50 mg/mL filter paper).

Results of Figure 3 show that the cellulases activity of the* Trichosporon *sp. yeast has a wide pH range, ranging from 3 to 9. The optimal pH of the CMCase was 5 (0.25 IU/mL), while that the FPase activity was maximal and stable (0.06 IU/mL) at pH varying from 4 to 7. The increase or the decrease of the pH values on either side of the optimal values was not followed by a rapid loss of activity, since the residual activities at pH 9 were 60% and 66.7%, respectively, for the CMCase and FPase activities. In addition, these enzymes retain at pH of 3 more than 40% and 26.7% of their activities, respectively (Figure 3). Thus, the cellulase enzymes produced by the yeast studied can be considered as tolerant to acidic and alkaline conditions. These results corroborate with those of Oikawa et al. [16] who showed that the yeast CMCase* Rhodotorula glutinis* KuJ is active at pH ranging from 2 to 7 with an optimal pH of 4.5. Geimba et al. (1999) have also shown that the CMCase of the fungus* Bipolaris sorokiniana* has a maximum activity at pH 5 but is inactive at pH of 3. The cellulases of the fungi* Aspergillus niger* and* Penicillium decumbens *[14] also have an optimal pH of 5.

### 3.5. Effect of Substrates Concentration on CMCase and FPase Activities

**Figure 4:**
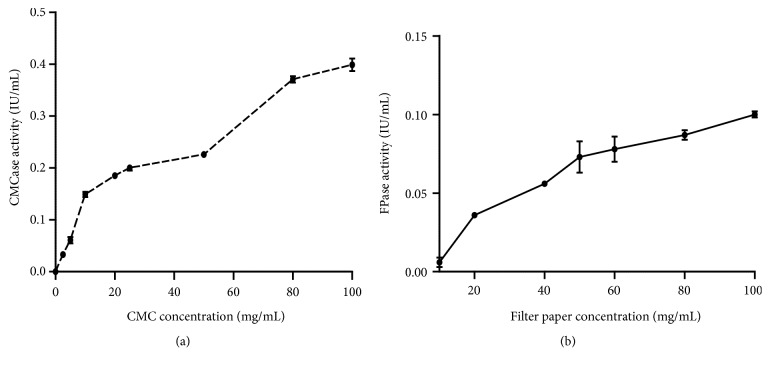
(a) Effect of CMC concentration on CMCase activity and (b) effect of filter paper concentration on FPase activity (pH 4.8; temperature 50°C).

The effect of the substrates concentration was also studied in order to optimize cellulase activities. The CMCase reaches a rate of 0.4 IU/mL at a concentration of 100 mg/mL of CMC and the FPase activity reaches 0.1 IU/mL at 100 mg/mL of the filter paper (Figures 4(a) and 4(b)). These results are in agreement with those of Saliu and Sani. [14], who showed that the CMCase activity of* Aspergillus niger *and* Penicillium decumbens* mushrooms was also maximal at a concentration of 100 mg/mL of CMC.

### 3.6. Kinetics of CMCase and FPase.

**Figure 5:**
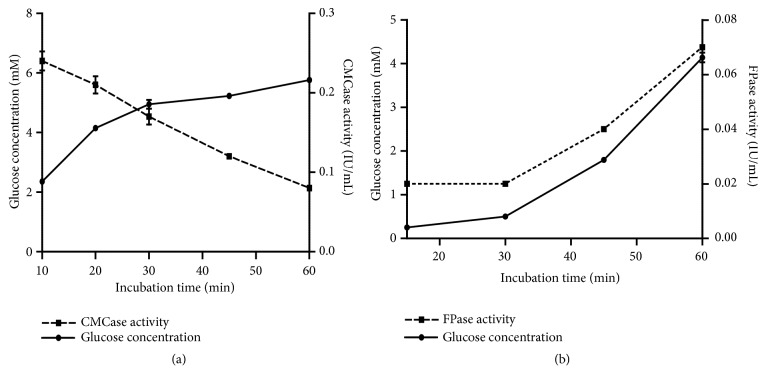
(a) Kinetics of CMCase (pH 4,8, temperature 50°C, and 10 mg/mL CMC). (b) Kinetics of FPase (pH 4.8, temperature 50°C, and 50 mg/mL filter paper).

The amount of the released glucose and the CMCase and FPase activities was determined using different incubation times. Results show in Figure 5(a) that the concentration of the released glucose increases with the incubation time in the presence of CMC from 10 to 30 min, and the maximum CMCase activity (0.24 IU/mL) was observed after 10 min of incubation. The best amount of the released glucose during the hydrolysis of the filter paper was 4.14 mM, after 60 min of incubation and the maximum FPase activity was 0.07 IU/mL (Figure 5(b)).

### 3.7. Optimization of Preincubation Time of the Filter Paper on FPase Activity

**Figure 6:**
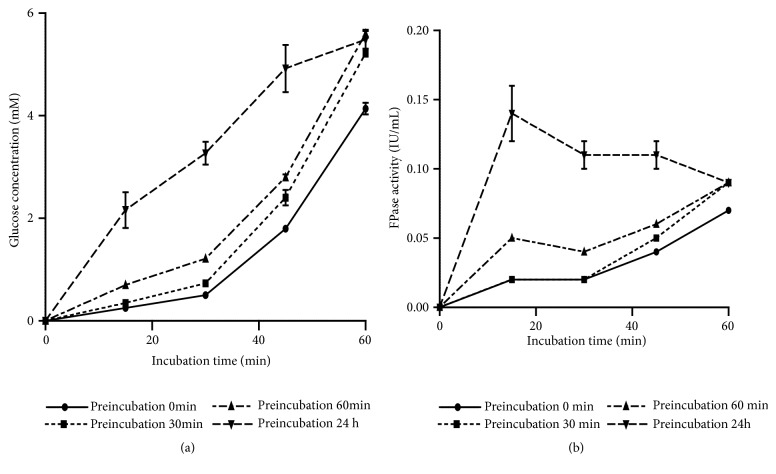
(a) Effect of preincubation time of the filter paper on glucose concentration; (b) the kinetics of FPase (pH 4.8, temperature 50°C, and 50 mg/mL filter paper).

Figure 6 demonstrates the presence of a 30 min phase where the FPase activity remains low. In order to reduce the latency time, preincubations of the filter paper in the buffer were performed at different times. When the preincubation time was 0, 10, 30, or 60 min, the best concentration of glucose (5.6 mM) and the highest FPase activity (0.1 IU/mL) was recorded only after 60 min. The best FPase activity (0.14 IU/mL) at 24-hour preincubation of the filter paper was obtained after 15 minutes. Hence, it was extremely interesting to apply the 24-hour preincubation of the filter paper in order to increase the FPase activity, which was probably due to a better absorption of the enzyme medium through the filter paper.

### 3.8. Evaluation of Cellulases Activities under Optimal Conditions

Results of the evaluation of cellulase activities under optimal conditions showed that the CMCase activity was 0.52 IU/mL at 10 min of incubation at temperatures of 55°C; the FPase activity was maximal (0.44 IU/mL) at 60°C, under 15 min of incubation (Table 1). The maximum hydrolysis activity of the cellulose fiber was about 0.56 IU/mL at 10 min at the temperatures of 65°C. The specific activity was 12.5, 11, and 14 U/mg of protein for CMCase, cellulase fiber, and FPase, respectively. The activities of these enzymes after optimization were three times higher for CMCase and five times with regard to FPase and cellulase fiber.

Industrial processes for the hydrolysis of cellulosic material into fermentable sugar are carried out under particular physical and chemical conditions, at the optimum values of the activity of available commercial enzymes. The yeast enzymes of this study are of considerable interest because of their activity at different temperatures and their ability to hydrolyze different soluble and insoluble cellulosic substrates. In fact, these enzymes may have potential applications in the treatment of agricultural waste and the bioremediation of cellulosic materials for sustainable bioethanol production.

## 4. Conclusion

In this study, 30 isolates of cellulosic yeasts were selected. The optimization of cellulase activities was performed on* Trichosporon *sp. genus. The results showed that this strain has a great potential to be used in the enzymatic saccharification of soluble (CMC) and insoluble (cellulose fiber and filter paper) cellulosic substrates at a wide pH range, with an optimum at 5, and at high temperatures of 55 to 70°C.

## Figures and Tables

**Table 1:** Optimal activities of cellulases enzymes (Acetate buffer pH 5, Substrate concentration; 50 mg/mL of CMC or cellulose Fiber and 80 mg/mL of filter paper).

Temperature (°C)	CMCase		FPase		Fibre cellulase	
	Activity (IU/mL)	Specific activity	Activity (IU/mL)	Specific activity	Activity (IU/mL)	Specific activity
		U/mg)		(U/mg)		(U/mg)
50	0.49±0.02	12.25±0.1	0.38±0.03	9.5±0.6	0.44±0.04	11±0.4
55	0.52±0.03	13±0.2	0.39±0.02	9.75±0.4	0.47±0.02	11.75±0.3
60	0.5±0.01	12.5±0.2	0.44±0.01	11±0.1	0.48±0.03	12±0.3
65	0.48±0.04	12±0.3	0.3±0.05	7.5±1.3	0.56±0.06	14±0.7
70	0.47±0.004	11.75±0.4	0.29±0.07	7.25±0.1	0.34±0.002	8.5±0.5

## Data Availability

The data used to support the findings of this study are available from the corresponding author upon request.
